# A Case of Replantation

**Published:** 1900-03-15

**Authors:** Frank R. Van Metre


THE
### DENTAL REGISTER.

Vol. LIV. MARCH 15, 1900. No. 3.

### A CASE OF REPLANTATION.

BY DR. FRANK R. VAN METRE.

Read before the Cincinnati Odontological Society, November 24, 1899.

It would seem egotistical, for one who has been in practice such a comparatively short time, to presume to give you a paper containing anything of material value. I shall, therefore, be content to report a case which I undertook some two and a half years ago, and endeavor to ask a few pertinent questions, in the hope of provoking discussion, from which we may possibly gain some valuable information.

The patient was a boy thirteen years of age. On May 15, 1897, he came to the office, with his superior right central in his pocket. Upon inquiry, I found this had been knocked out by a base ball, the blow being on the lip and sufficiently high to have forced out the tooth, without breaking it in any way. The lip was badly swollen, but, strange to say, no cut or split was visible. The alveolar process was not shattered, although badly bruised tissue gave evidence of a blow of much force. The remaining central was intact and firm, although the shock had made it more or less sensitive. The right lateral had not fared so well, and was not only quite loose, but had been forced somewhat from its socket. The cuspid on this side was just erupting, and lay well over the loosened lateral, seemingly determined to finish the job by forcing it out.

The accident occurred two hours or more before the case presented. When he handed me the central, I found it not only cold but absolutely dry on the outer surface, and quite dirty from handling. It was free from decay, and also free from any check which one might expect to find after such a telling blow.

I removed the coating of rich earth (such as one finds out in the Mill Creek bottoms). The pulp was not molested by me. After wrapping the tooth in 10 percent iodoform gauze, I turned my attention to the socket. The cavity was not bleeding, but I found it clogged with clotted blood and bruised tissue. This was carefully removed and the socket washed out, after which the tooth was replanted and secured to the firm central by means of ligatures, which were worn for two weeks, in which time the soreness was much reduced and the tooth firm enough to allow of an impression being taken, which was done, and the replanted tooth anchored to the sound one by means of gold bands. In two more weeks the cuspid had sufficiently erupted to retain a cap, which was placed on it at once, the molar banded and an appliance put on to draw the cuspid back so as to clear the loose lateral which it seemed determined to crowd out.

After the teeth had gotten in a fair way to recovery, they were both again loosened by an elbow jab as he was going up the steps with a crowd of school companions. This caused extra work and much more time, however I removed the bands on the first of October, five and one half months after the injury. The parts were quite firm and the soreness entirely gone.

The replanted tooth undoubtedly contains a lifeless pulp. It is slightly darker than its fellows, but gives no inconvenience, and is subject to the same usage as the rest.

After two and a half years, I find it still firm, never having given the slightest inconvenience since the removal of the appliances.

Now, what union has taken place to hold it so perfectly solid?

Why has the devitalized pulp not abscessed?

What is the probable "life" of such an operation?

How much better would it be to have removed the pulp and filled canal before replanting?

Would chances for recovery be as fair in a patient from forty to fifty years old?

The blow which so loosened the teeth two weeks after the first operation was certainly not conducive to best results, yet the case cleared up satisfactorily, even in the face of all this. Then why would not transplantation be practicable in many cases where extraction is necessary?

Why is it not oftener resorted to?

DISCUSSION OF DR. VAN METRE'S PAPER.

DR. FLETCHER: One of my earliest remembrances of the wonderful things in dentistry was my preceptor's replacing, for a young lady of about twenty-five, a lateral incisor which had been driven from its socket by a fall on the ice. The tooth was out several hours. Nothing by way of filling pulp canal was done. To my knowledge it remained in its place after being replanted, three years.

DR. CASSIDY: It has been asked which is the better procedure—to devitalize the pulp in these cases, or not, before replacing. I should prefer to devitalize, otherwise the tooth would merely be tolerated.

DR. H. A. SMITH: If there is anything which will justify replantation it is traumatism, or accident. The life of such replanted teeth has been found to be about five years; say five to seven years. It is a characteristic of a replanted tooth that up to the very last it will remain quite firm in its socket, then will suddenly loosen and fall out. A curious inquiry arises, what becomes of the pulp of the tooth in these cases? If the tooth is finally lost by absorption what happens to the pulp? The evidence is that it has become devitalized, but why then has there not been the usual sequel of abscess? In some cases, it is true, we do have abscess.

This operation is not now frequently attempted, since we have become familiar with the researches of Lister—since we have so advanced in bacteriological investigations—and this despite the fact that to-day we are much better able to control all adverse conditions. This subject of replantation, implantation, transplantation—in all its varied phases—comes up from time to time, every ten years or so, in our dental meetings.

Drs. Kumler, Stacy, Locke and Rauh cited cases of replantation in their practices or coming under their observation. Dr. Fletcher stated that years ago he instituted a series of experiments, implanting the teeth of a dog in the leg bone of a goat, for the purpose of studying the phenomena of tissue formation, absorption, resorption, and other attending processes. His conclusion after long and careful investigation was that nothing in the natural processes of repair, under such conditions as there noted, encourages a deliberate choice of this method of repairing a loss of the nature referred to in the paper read.

Dr. Molyneaux: I have always attached importance to the manner of manipulation in these cases, as an element of success. I recall seeing my father re insert several teeth which had been knocked out by an accident, when I was a boy. A young married couple had been all mashed up in a street-car accident. He folded the three or four teeth which had been knocked out in a napkin saturated with salt and water solution, and the case turned out very favorably. Since that day I have always felt that I stood a better chance of success in similar cases, if I followed his example. I do not believe in decrying the operation, even if we have no assurance of permanency of retention of the re-inserted teeth. If they last only a few years, I assume that the patient has been well served.

